# Allogeneic stem cell transplantation in acute lymphoblastic leukemia patients older than 60 years: a survey from the acute leukemia working party of EBMT

**DOI:** 10.18632/oncotarget.22934

**Published:** 2017-12-04

**Authors:** Gabrielle Roth-Guepin, Jonathan Canaani, Annalisa Ruggeri, Myriam Labopin, Juergen Finke, Jan J. Cornelissen, Jeremy Delage, Gernot Stuhler, Monserrat Rovira, Mike Potter, Michael Stadler, Hendrik Veelken, Jean Yves Cahn, Matthew Collin, Yves Beguin, Sebastian Giebel, Arnon Nagler, Mohamad Mohty

**Affiliations:** ^1^ Hematology Department, CHU Brabois, Nancy, France; ^2^ Hematology Division, Chaim Sheba Medical Center, Tel Aviv University, Tel-Aviv, Israel; ^3^ Acute Leukemia Working Party–EBMT and Department of Hematology and Cell Therapy, HÔpital Saint-Antoine, Paris, France; ^4^ INSERM UMR 938, Paris, France; ^5^ Université Pierre et Marie Curie, Paris, France; ^6^ Department of Hematology and Oncology, University Medical Center Freiburg, Freiburg, Germany; ^7^ Erasmus MC Cancer Institute, University Medical Center Rotterdam, Department of Hematology, Rotterdam, The Netherlands; ^8^ Service d’Hématologie et d’Oncologie, Hospital Lapeyronie CHU , University Montpellier, Montpellier, France; ^9^ Deutsche Klinik für Diagnostik, KMT Zentrum, Wiesbaden, Germany; ^10^ Hospital Clinic Barcelona, Institute of Hematology and Oncology, Department of Hematology, Barcelona, Spain; ^11^ Institute of Hematology and Oncology, Department of Hematology, London, UK; ^12^ Department of Hematology, Hemostasis, Oncology and Stem Cell Transplantation, Hannover, Medical School, Hannover, Germany; ^13^ Department of Hematology, Leiden University Medical Center, Leiden, Netherlands; ^14^ Department of Hematology, University Hospital, Grenoble, France; ^15^ Newcastle University, Adult HSCT unit, Northern Centre for Bone Marrow Transplantation, Freeman Hospital, Newcastle upon Tyne, UK; ^16^ University of Liege, Department of Hematology, CHU of Liège, Liège, Belgium; ^17^ Department of Bone Marrow Transplantation and Oncohematology, Maria Sklodowska-Curie Memorial Cancer Center and Institute of Oncology, Gliwice, Poland

**Keywords:** acute lymphoblastic leukemia, allogeneic hematopoietic cell transplantation, graft versus host disease, elderly, cytomegalovirus

## Abstract

Hematopoietic stem cell transplantation (HSCT) is being increasingly explored as a treatment modality for older patients with acute lymphoblastic leukemia (ALL). Yet, concerns regarding the long term outcome of transplantation in older patients limit the wide spread applicability of this approach. In this analysis we set out to determine the outcome of ALL patients over the age of 60 who underwent reduced intensity HSCT. Herein, we present the experience of the acute leukemia working party (ALWP) of the EBMT in this age group. We analyzed a cohort of 142 patients transplanted in first remission with a median age of 62 (range 60–76 years) and a median follow-up period of 36 months post-transplant. At 3 years, overall survival (OS) and leukemia-free survival were 42% and 35%, respectively. Multivariate analyses identified cytomegalovirus (CMV) donor-recipient matching (CMV D+/R+) to be significantly associated with inferior OS. Patients transplanted from unrelated donors experienced increased grade II-IV acute graft versus host disease compared to those receiving grafts from matched related donors [Hazard ratio (HR) of 3.7, 95% confidence interval (CI), 1.75–7.8; *p* = 0.0005). Outcome was not impacted by Philadelphia chromosome status. A select subset of older ALL patients will benefit from extended survival and a disease free state following HSCT.

## INTRODUCTION

The introduction of reduced intensity (RIC) allogeneic stem cell transplantation into the mainstay of clinical management of older patients with acute lymphoblastic leukemia (ALL) more than a decade ago [1] represented a significant paradigm change for clinicians treating this challenging segment of the acute leukemia field. As is the case for younger ALL patients, an allogeneic stem cell transplant endeavors to provide older patients with the best possibility for disease control, while remaining cognizant of the overarching principals of therapy for this unique patient population, namely minimizing treatment related toxicity and mortality [1–3]. Whereas standard chemotherapy based approaches have yielded for the most part disappointing results in this age group with long term survival of only 6–21% [4–8], transplant is increasingly being used in an attempt to improve on these suboptimal results. Yet, the benefit of transplantation in this age group is not as evident as that seen for younger patients, for example those at the 18–39 year age group where 5 year overall survival rates of 66% surpass by far the reported 22% survival rate for patients 60–69, as published recently by the Dutch cancer registry [9]. Moreover, given the substantial transplant related mortality and morbidity reported by several groups [2, 10–13], it remains unclear which patients derive the most benefit from a transplant based approach. Thus, defining specific patient subsets who would specifically benefit from transplantation is of utmost importance for patients and clinicians alike. Using the multicenter registry of the acute leukemia working party (ALWP) of the European Society of Blood and Marrow Transplantation (EBMT) we provide a current assessment of the clinical landscape for older ALL patients over the age of 60 undergoing allogeneic stem cell transplantation after a RIC regimen.

## RESULTS

### Patient, disease and transplant characteristics

In all, 142 patients were identified and their baseline clinical and laboratory data are summarized in Table 1. The median age of patients on the cohort was 62 (range 60–76 years) with a median follow-up period of 36 months (range 2–123 months). Notably, a little more than half of the cohort analyzed was Philadelphia chromosome positive. Fludarabine based regimens were the most frequently used approaches for conditioning prior to transplant whereas PB was the most commonly used source for stem cell grafts (135 patients; 95%). Donor was matched sibling for 66 patients, and 10/10 HLA matched unrelated donors for 76 patients. Of note, most Philadelphia chromosome positive patients were treated with tyrosine kinase inhibitors (TKI) prior to transplant (45 patients; 70%), and 20 patients received TKI post-transplant indicated either for prophylaxis (7 patients), positive minimal residual disease (MRD) studies (5 patients) or because of relapse (8 patients).

**Table 1:** Baseline characteristics of study population

Parameter	*N* = 142
Follow up duration in m, median (range)	36 (1.8–123.4 )
Age in years, median (range)	62 (60–76)
Gender, *n* (%)	
Male	65 (45.7)
Female	77 (54.2)
ALL type	
B-ALL	126 (88.7)
T-ALL	11 (7.7)
Other	5 (3.5)
Philadelphia Chromosome	
Ph negative	59 (41.5)
Ph positive	83 (58.4)
Donor type, *n* (%)	
Identical sibling	66 (46.4)
Matched unrelated	76 (53.5)
Stem cell source, *n* (%)	
Peripheral blood	135 (95)
Bone marrow	7 (4.9)
Conditioning regimen, *n* (%)	
Flu/Mel	28 (19.7)
Flu/Bu	58 (40.8)
Cy/Thio	1 (0.7)
Flu/Treo	2 (1.4)
Bu/Clo	6 (4.2)
Cy/Flu	3 (2.1)
Flu/Thio/BCNU	11 (7.7)
Cy/Treo	1 (0.7)
TBI	32 (22.5)
Donor-recipient CMV match, *n* (%)	
CMV D−/R−	33 (23.4)
CMV D+/R−	13 (9.2)
CMV D−/R+	39 (27.6)
CMV D+/R+	56 (39.7)
Missing	1
Karnofsky score at transplant	
< 90	45 (33.8)
≥ 90	88 (66.1)
Missing	9
HCT-CI	
0	41 (42.7)
1–2	25 (26)
≥ 3	30 (31.2)
Missing	46

Abbreviations: CMV, cytomegalovirus; Flu/Mel, fludarabine and melphalan; Flu/Bu, fludarabine and busulfan; Cy/Thio, cyclophosphamide and thiotepa; TBI, total body irradiation; HCT-CI, Hematopoietic Stem Cell Transplant Comorbidity Index.

### Acute and chronic GVHD

The cumulative incidence of grade II-IV acute GVHD at 100 days was 29% (95% CI: 21%–37%) while the incidence of grade III-IV acute GVHD was 9% (95% CI: 5%–15%). As shown in the univariate analysis presented in Supplementary Table 1, patients transplanted from matched unrelated donors had significantly worse grade II-IV acute GVHD rates compared to patients transplanted from matched sibling donors (39% versus 16%; *P* = 0.001). As detailed in the multivariate analysis shown in Table 2, patients transplanted from URD experienced increased grade II-IV acute GVHD compared to patients receiving grafts from MSD (HR = 3.7, 95% CI, 1.75–7.8; *P* = 0.0005). Conversely, CMV D+/R+ status was also associated with a trend towards a higher rate of grade II-IV acute GVHD (HR = 1.79, 95% CI, 0.93–3.41; *P* = 0.07).

**Table 2:** Multivariate analysis of factors impacting on clinical outcome

Outcome	Hazard ratio (95% CI)	*P*
**RI**		
URD versus MSD	0.72 (0.39–1.31)	0.28
Philadelphia chromosome positive ALL	0.64 (0.35–1.15)	0.14
**NRM**		
CMV D+/R+ versus others	2.25 (1.09–4.66)	0.028
**LFS**		
CMV D+/R+ versus others	1.62 (1.01–2.6)	0.045
**OS**		
CMV D+/R+ versus others	1.78 (1.09–2.89)	0.02
**GRFS**		
CMV D+/R+ versus others	1.33 (0.89–1.99)	0.15
**Grade II-IV acute GVHD**		
URD versus MSD	3.7 (1.75–7.8)	0.0005
CMV D+/R+ versus others	1.79 (0.93–3.41)	0.07

Abbreviations: CR, complete remission; GVHD, graft versus host disease; GRFS,GVHD-free/relapse-free survival; CMV, cytomegalovirus; URD, unrelated donor; MDS, matched sibling donor.

### RI and NRM

At 3 years the cumulative incidence of relapse was 40% (95% CI: 32%–48%) whereas the rate of NRM at 3 years was 23% (95% CI: 16%–30%). With univariate analysis (Supplementary Table 2), we observed that in male patients receiving grafts from female donors there was a trend suggesting increased relapse rates (61% versus 36%; *P* = 0.072). In multivariate analysis, RI was not significantly affected by donor source nor by Philadelphia chromosome positivity (Table 2). NRM was significantly affected by the CMV status in both donor and patient whereby D+/R+ patients experienced increased NRM rates (HR = 2.25, 95% CI, 1.09–4.66; *P* = 0.028).

### GRFS, LFS and OS

**Figure 1:**
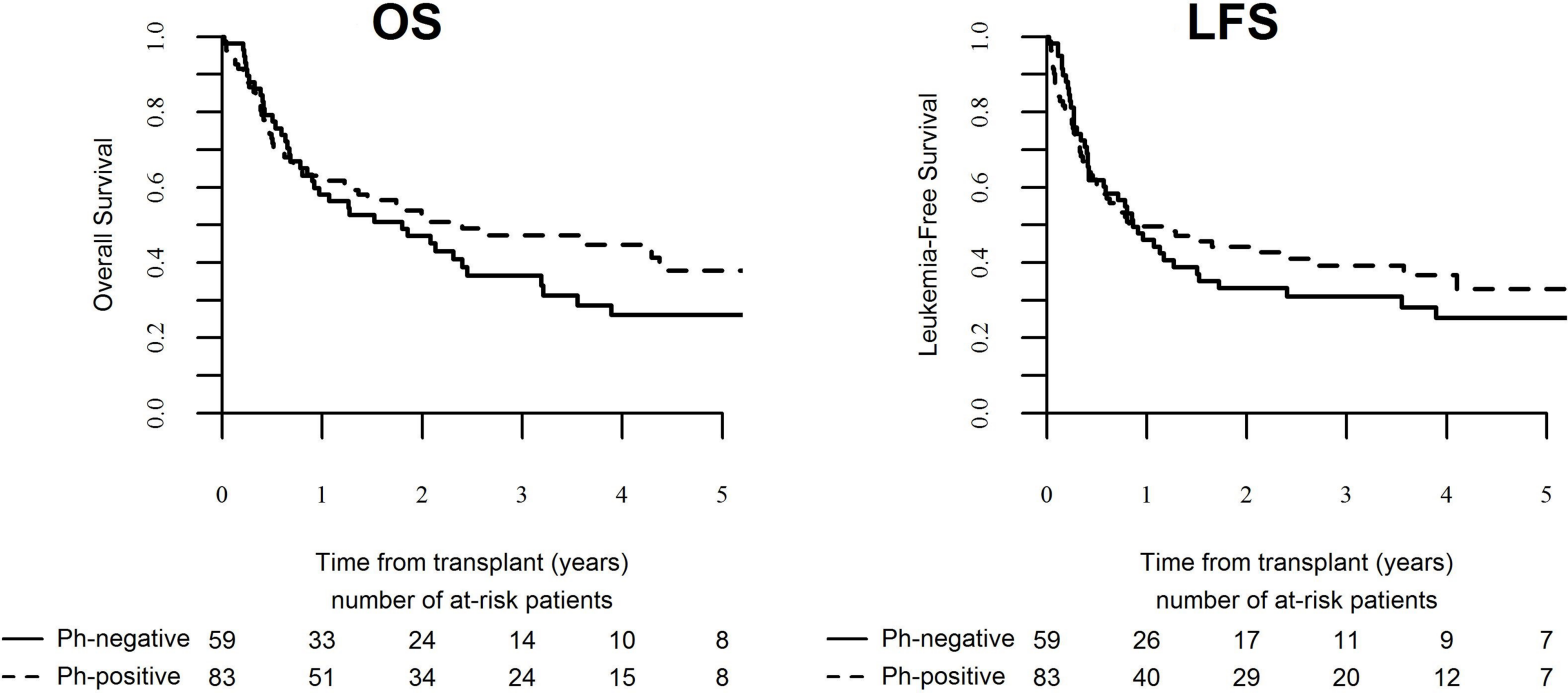
Outcome of ALL patients over the age of 60 following transplantation in CR1 per Philadelphia chromosome status (**A**)- OS, (**B**)- LFS.

Patients in the analyzed cohort had 3 year OS and LFS rates of 42% and 35% respectively. The most frequent causes of death in the analyzed cohort were leukemia relapse (41 patients; 49%), infection (18 patients, 21%), and GVHD (14 patients, 16%). Results of a univariate analysis are summarized in Supplementary Table 1. In multivariate analysis, LFS and OS rates were significantly influenced by CMV D+/R+ status whereby CMV D+/R+ (HR = 1.62, 95% CI, 1.01–2.6; *P* = 0.045, and HR = 1.78, 95% CI, 1.09–2.89; *P* = 0.02) conferred inferior rates for both clinical indices. The GRFS rate was not significantly impacted by any of the abovementioned factors. Philadelphia chromosome status did not significantly impact on clinical outcome (Figure 1).

## DISCUSSION

Older patients with ALL are a uniquely challenging patient population where clinicians are faced with the need to maintain a finely tuned balance between treating leukemia optimally while minimizing the attendant treatment related toxicity. In this analysis we present our recent experience with a large group of older ALL patients undergoing transplant with a curative intent. Our data indicate that a significant subset of patients will be alive and disease free at 3 years following transplant (42% and 35%, respectively), furthermore we show that the traditionally designated high risk Ph^+^ patients experience equivalent clinical outcome to Ph^-^ patients in terms of both leukemia control and transplant associated toxicity.

While outcome of adult patients as a whole has decidedly improved over the past decade [14], selection of the older ALL patient most suitable for undergoing HSCT remains a difficult and challenging clinical decision. Indeed, this is reflected in a wide variation in practice, where for example a recent survey conducted among Canadian hematologists revealed that only half of them would recommend upfront transplant for ALL patients over the age of 35 [15] while on the other hand published data from the Netherlands indicates that 19% of patients between the ages of 60–69 were transplanted between the years 2007–2012 [9]. The justified concerns of transplant related morbidity and mortality in the inherently vulnerable population of older patients must be contrasted with the realization of the poor outcome associated with non-transplant approaches yielding a five year overall survival rate of only 21% for non-transplanted adults over the age of 55 as observed for instance in the MRC UKALL XII/ECOG E2993 [6, 16]. In our current analysis we found that older patients attained survival rates of more than 40%, in agreement with recently published data in older ALL patients [17, 18], and probably in concurrence also with the MRC UKALL XII/ECOG E2993 [6] and the Japanese experience [19] which evaluated survival at different time points (5 and 2 years respectively).

Our results confirm that the transformative role of TKI in Ph^+^ ALL also extends to the segment of older Ph^+^ patients undergoing transplantation. Indeed, our data suggests that for older patients with Philadelphia chromosome positive ALL, prognosis equates with that of Philadelphia chromosome negative patients. Accordingly, it may possible to consider that in older Ph^+^ patients fit for transplant, Philadelphia chromosome status no longer designates patients as having higher risk disease, a designation which was based on the historical poor outcomes in this patient population [20, 21].

Data from our group [22] and others [13, 17, 23] have established the non-inferiority of RIC and myeloablative conditioning in terms of relapse incidence. Yet, the optimal RIC regimen has yet to be determined. Most of the centers in our cohort used reduced intensity fludarabine based regimens with either melphalan or busulfan, a pattern shared with the experience reported by others [11, 18, 19]. Kanamori and colleagues suggested that fludarabine/busulfan was superior to fludarabine/melphalan although their results did not reach statistical significance [19]. As of now, no data exist to demonstrate a definitive advantage in using a given conditioning regimen. Our retrospective study was not designed to assess the differences between the various conditioning regimens and thus the optimal conditioning regimen to use in this setting remains an open question.

An unexpected finding in our analysis pertained to the clinical impact of CMV donor-recipient matching. We found CMV D^+^/R^+^ to be significantly associated with inferior overall survival and leukemia free survival coupled with increased non-relapse mortality. In an earlier EBMT analysis from the 1990s [24] looking at a large cohort of acute leukemia and chronic myeloid leukemia patients, those patients receiving grafts from CMV-seropositive HLA-identical sibling donors had the same survival as patients grafted from seronegative donors. Notably, in that study patients receiving unrelated donor grafts from CMV-seropositive donors had an improved 5-year survival. This issue was recently readdressed with data showing that for patients undergoing myeloablative conditioning, CMV D^+^/R^+^ donor-recipient status correlated with improved overall survival compared with CMV D^-^/R^+^. This effect was absent in the reduced-intensity conditioning cohort of the analysis [25]. Of note no distinction was made in this study between ALL and AML. A recently published analysis from the EBMT [26] on 5158 ALL patients seem to be in line with our data, indicating that CMV seropositivity of the donor and/or the recipient is significantly associated with decreased 2-year leukemia-free survival and overall survival, and increased non-relapse mortality. Why donor-recipient CMV matching would impact significantly on clinical outcome is still unclear, though some preclinical observations suggest that CMV infection modulates the NK cell repertoire following transplantation thus impacting on acute myeloid leukemia relapse [27].

Owing to the multicenter retrospective nature of our analysis, interpretation of our results needs to be undertaken cautiously. Several factors which were not captured by our registry, including measurable residual disease (MRD) data as well as depth of molecular remission (in Philadelphia chromosome positive patients) prior to transplant, would undoubtedly would have further informed our analysis. In addition, we acknowledge that the cohort analyzed was a select group of patients fit for transplant which may not be completely characteristic of the complete older ALL patient population.

## MATERIALS AND METHODS

### Study design and data collection

This is a retrospective multicenter analysis based on the registry data of the ALWP of the EBMT. The EBMT is a voluntary working group comprising more than 500 transplant centers that are required to report all consecutive stem cell transplantations and follow-ups once a year. Audits are routinely performed to determine the accuracy of the data. This study was approved by the ALWP institutional review board. The study was conducted in accordance with the Declaration of Helsinki and Good Clinical Practice guidelines. All patients provided written informed consent authorizing the use of their personal information for research purposes. Eligibility criteria for this analysis included adult ALL patients over 60 years of age who underwent RIC HSCT in first remission between 2005 and 2014. Intensity of conditioning was determined according to published criteria [28]. Stem cell graft consisted of either bone marrow (BM) or G-CSF mobilized peripheral blood (PB). All donors were HLA-matched according to standard criteria (locus-A, -B, -C, DRB1, -DQB1). Exclusion criteria were: previous allogeneic, cord blood or haploidentical transplantation, *ex vivo* T cell depleted stem cell graft. Comorbidity scores were evaluated by the modified EBMT score [29] and the HCT-CI [30]. Grading of acute GVHD and chronic GVHD was performed using established criteria [31, 32].

### Statistical analysis

Clinical outcomes were evaluated as follows: (i) non-relapse mortality (NRM), defined as death without previous relapse; (ii) relapse incidence (RI), defined on the basis of morphological evidence of leukemia in bone marrow or other extramedullary organs; (iii) leukemia-free survival (LFS), defined as the time from transplantation to first event (either relapse or death in complete remission); (iv) GVHD-free/relapse-free survival (GRFS), defined as events including grade 3–4 acute GVHD, extensive chronic GVHD, relapse, or death in the first post-HCT year [33]; and (v) overall survival. Cumulative incidence curves were used for RI and NRM in a competing risks setting, since death and relapse are competing. Probabilities of OS, LFS, and LFS were calculated using the Kaplan–Meier estimate. Univariate analyses were done using the Gray's test for cumulative incidence functions and the log rank test for OS, GRFS, and LFS. Multivariate analyses were performed by stepwise selection of variables associated with *p* < 0.15 in univariate analysis. All tests were two-sided with the type I error rate fixed at 0.05. Statistical analyses were performed with SPSS 19 (SPSS Inc, Chicago, IL, USA) and R 2.13.2 (R Development Core Team, Vienna, Austria) software packages.

## CONCLUSIONS

Taken as a whole, our findings indicate that allogeneic stem cell transplantation is older patients with ALL is feasible and offers the possibility of a long term disease free state in some patients regardless of Philadelphia chromosome status. Patients in first remission, with matched sibling donors, and with CMV donor-recipient matching other than CMV D^+^/R^+^ probably fare better than other patients. As we expect novel therapeutics to form the centerpiece of management of ALL patients in the near future, it is cautiously anticipated and hoped that the outcome of older patients with ALL will further improve.

## SUPPLEMENTARY MATERIALS TABLES



## Abbreviations

ALL: Acute lymphoblastic leukemia
EBMT: European Society for Blood and Marrow Transplantation
ALWP: Acute leukemia working party
GVHD: Graft versus host disease
GRFS: GVHD-free/relapse-free survival
LFS: Leukemia free survival
OS: Overall survival
RI: Relapse incidence
