# Supplementary material for: Allogeneic stem cell transplantation in acute lymphoblastic leukemia patients older than 60 years: a survey from the acute leukemia working party of EBMT
Source: Oncotarget. 2017 Dec 4;8(68):112972–9. doi: 10.18632/oncotarget.22934 (PMC5762566; doi:10.18632/oncotarget.22934)
Supplement: Supplementary file 1 [file oncotarget-08-112972-s001.pdf]

Allogeneic stem cell transplantation in acute lymphoblastic leukemia patients older than 60 years: a survey from the acute leukemia working party of EBMT

SUPPLEMENTARY MATERIALS

Supplementary Table 1: Univariate analysis of factors impacting on transplant related outcome

Parameter	GRFS	*P*	Acute GVHD II-IV	*P*	Chronic GVHD	*P*
Gender						
Male	12.6% [3.9–21.3]	0.1	29.8% [19–41.3]	0.86	43.5% [30.6–55.7]	0.15
Female	26.8% [16.6–37.1]		28.8% [18.8–39.6]		33.1% [22.5–44.2]	
Donor						
Matched sibling donor	17.7% [7.8–27.5]	0.61	16.8% [8.5–27.4]	0.001	33.5% [22–45.4]	0.42
Matched unrelated donor	21.8% [11.9–31.8]		39.7% [28.6–50.6]		41.6% [29.8–52.8]	
Karnofsky score						
< 90	26.7% [13.7–39.6]	0.31	33.5% [20.1–47.5]	0.27	31.1% [18.1–45]	0.32
≥ 90	15.5% [6.7–24.2]		25.1% [16.3–34.9]		42.1% [31–52.9]	
CMV status Donor-Recipient						
D-/R-	24.8% [9.3–40.2]	0.29	33.9% [18.1–50.3]	0.58	19.1% [7.5–34.8]	0.083
D+/R-	38.5% [12–64.9]		16.1% [2.3–41.4]		46.2% [16.7–71.6]	
D-/R+	19.8% [6.6–33]		23.4% [11.5–37.8]		50.1% [32.9–65.1]	
D+/R+	12.8% [2.9–22.7]		33.9% [21.4–46.8]		38.5% [25.1–51.8]	
Philadelphia chromosome						
Negative	13.2% [3.9–22.5]	0.42	29.1% [18–41.1]	0.93	39.4% [26.4–52.2]	0.66
Positive	24.4% [14.5–34.4]		29% [19.4–39.3]		36.8% [26–47.5]	

Abbreviations: CMV, cytomegalovirus; GVHD, graft versus host disease.

Supplementary Table 2: Univariate analysis of factors impacting on clinical outcome

Parameter	3 year RI	*P*	3 year NRM	*P*	3 year LFS	*P*	3 year OS	*P*
Philadelphia Chromosome								
Negative	50.9% [37–63.1]	0.09	18.1% [9.2–29.5]	0.29	31% [18.7–43.3]	0.55	36.5% [23.5–49.5]	0.31
Positive	33.3% [23.2–43.7]		27% [17.5–37.5]		39.3% [28.2–50.3]		47.2% [35.8–58.7]	
Donor								
Matched sibling donor	46.8% [33.9–58.6]	0.13	22.8% [13.1–34.2]	0.56	29.8% [18.2–41.5]	0.23	38.6% [26.2–51]	0.73
Matched unrelated donor	35.2% [24.4–46.1]		23.6% [14.4–34.1]		41.3% [29.8–52.7]		46.3% [34.3–58.3]	
Karnofsky score								
<90	31.1% [18.2–44.9]	0.13	28.9% [16.4–42.6]	0.08	40% [25.7–54.3]	0.63	45.8% [31–60.7]	0.88
≥90	43.6% [32.7–53.9]		20.2% [12–30]		35.6% [24.9–46.4]		44% [32.9–55.2]	
Patient gender								
Male	47.2% [34.3–59]	0.19	22.6% [13–33.8]	0.52	30.3% [18.7–41.9]	0.38	37% [24.7–49.4]	0.4
Female	34.7% [24–45.6]		23.9% [14.6–34.5]		41% [29.5–52.5]		47.5% [35.5–59.6]	
Donor-recipient gender matching								
No F ->M	36.2% [27.5–44.9]	0.072	25.1% [17.4–33.6]	0.15	38.4% [29.2–47.5]	0.59	44.3% [34.9–53.8]	0.76
F ->M	61.7% [35.7–79.7]		14.9% [3.2–34.9]		23.4% [4.3–42.4]		30.7% [9.5–51.9]	
In-vivo T cell depletion								
No	35.9% [21.9–50.2]	0.26	26% [13.5–40.2]	0.85	38.1% [23.1–53.2]	0.27	46.3% [30.7–62]	0.59
Yes	42.9% [32.7–52.8]		22.1% [14.1–31.2]		34.5% [24.7–44.3]		40.9% [30.5–51.2]	
TKI use in Ph+ ALL								
No	42.1% [19.5–63.2]	0.22	28.4% [9.3–51.4]	0.61	29.5% [7.8–51.1]	0.28	37.9% [13.4–62.4]	0.35
Yes	24.4% [13–37.8]		28.2% [15.3–42.6]		47.3% [32.2–62.5]		53.8% [38.6–68.9]	

Abbreviations: TKI, tyrosine kinase inhibitor; RI, relapse incidence; NRM, non-relapse mortality; LFS, leukemia free survival; OS, overall survival.
